# Acceptability of a clofazimine tablet in children with rifampicin-resistant TB in three high-burden countries

**DOI:** 10.5588/ijtldopen.25.0309

**Published:** 2025-08-13

**Authors:** L. Viljoen, H.R. Draper, N.T. Castillo-Carandang, N. Suryavanshi, A. Marthinus, A.M.A. Cheong, J.D.D. Ocampo, G. Dhumal, S. Bagchi, D.T. Wademan, A. Kinikar, M. Paradkar, M.V.G. Frias, D.J.O. Casalme, A.C. Hesseling, A.J. Garcia-Prats, M. Palmer, G. Hoddinott

**Affiliations:** ^1^ Desmond Tutu TB Centre, Department of Paediatrics and Child Health, Faculty of Medicine and Health Sciences, Stellenbosch University, Cape Town, South Africa;; ^2^ Department of Clinical Epidemiology, College of Medicine, University of the Philippines Manila, Philippines;; ^3^ De La Salle Health Sciences Institute, Dasmariñas City, Cavite, the Philippines;; ^4^ Program on Social Innovation in Health, National Institutes of Health, University of the Philippines Manila, Philippines;; ^5^ Byramjee Jeejeebhoy Government Medical College -Johns Hopkins Clinical Research Site, Pune, India;; ^6^ Department of Paediatrics, Byramjee Jeejeebhoy Government Medical College, Pune, India;; ^7^ Department of Pediatrics, School of Medicine and Public Health, University of Wisconsin, Madison, Wisconsin, USA;; ^8^ School of Public Health, Faculty of Medicine and Health, The University of Sydney, Sydney, Australia.

**Keywords:** tuberculosis, drug-resistant TB, dispersible tablets, South Africa, India, Philippines

## Abstract

**BACKGROUND:**

Rifampicin-resistant TB (RR-TB) in children is frequently treated with clofazimine (CFZ), widely available as a 100mg gel capsule. This formulation is challenging to administer and is poorly acceptable to children and caregivers. Poor acceptability may negatively impact adherence and treatment outcomes. We describe the acceptability of a novel 50mg CFZ tablet formulation among children in South Africa, India, and the Philippines.

**METHODS:**

Mixed methods assessments were completed in a moxifloxacin and CFZ safety and pharmacokinetics trial in children with RR-TB. Quantitative data were collected from 36 participants at 4 timepoints. A sub-sample of 26 child/caregiver dyads participated in ∼4 qualitative interviews. Descriptive statistics and thematic analysis were employed.

**FINDINGS:**

The median age of n=36 participants (South Africa n=20; India n=6; the Philippines n=10) was 4.9 years. The majority (29/36) received a CFZ gel capsule prior to switching to the tablet formulation. The 50mg tablet had better acceptability scores for taste (p=0.035), smell (p=0.035), and ease of swallowing (p=0.02) compared to gel capsules. Participants described the tablet formulation as easier to administer/take without a lingering smell or taste. Limited concerns were noted on staining.

**CONCLUSION:**

The novel 50mg CFZ tablet has better acceptability and should be prioritised for children wherever possible.

At the 2023 United Nations high-level meeting on the fight against TB, member states committed to providing life-saving treatment that is acceptable for people (including children) with drug-susceptible (DS) and drug-resistant TB (DR-TB).^1^ Rifampicin-resistant TB (RR-TB) is caused by *Mycobacterium tuberculosis* resistant to rifampicin, with or without resistance to other first-line anti-TB drugs, and multidrug-resistant TB (MDR-TB) is resistant to at least isoniazid and rifampicin.^2^ Approximately 410,000 people globally, including 25,000 to 32,000 children <15 years old, were estimated to develop RR-TB or MDR-TB in 2022 alone.^3,4^ While RR-TB is a global health concern, eight countries (including India, the Philippines, and South Africa) account for two-thirds of all cases.^5^ Treatment for RR-TB is associated with biologically adverse effects including nausea, insomnia, dizziness, headaches, vomiting, hepatotoxicity, cardiac rhythm abnormalities (QT prolongation), and abdominal pain.^6^ More broadly, the experience of RR-TB and its treatment is often negative, with multiple compounding layers of economically, socially, and psychologically deleterious effects.^7^ Recent developments in novel and repurposed TB drugs, improved regimens, and better formulations have led to reductions in RR-TB treatment duration and the frequency and severity of some negative consequences.^8^ Still, child-friendly formulations are needed for RR-TB to ensure effective, safe, and acceptable administration of treatment.^9^

Clofazimine (CFZ) is frequently used in RR-TB treatment regimens, including for children. However, it is associated with skin hyperpigmentation, gastrointestinal irritation, and QT prolongation.^10,11^ The most widely available CFZ formulation is a 100mg gel capsule, which is notably unpalatable and challenging to administer to young children.^10^ For children who require <100mg doses, manipulation of this formulation is required, and some caregivers resort to extracting the liquid from the capsule with a syringe for oral administration. This process is both inaccurate for dosing and difficult since the liquid stains surfaces (including clothes and furniture) orange.^12^ An alternate approach in young children that avoids some of these dosing challenges, is to prescribe the 100mg capsule on alternate days or three times per week. This schedule is, however, complicated for children, their caregivers, and health workers, and impacts drug concentrations that could affect efficacy and safety.^10,13^

Stakeholders, including community and advocacy groups, have called for TB treatment formulations for children that are more acceptable to end-users.^14^ We assessed the acceptability of a novel 50mg CFZ solid tablet (Macleods Pharmaceuticals, Mumbai, India), which, although not marketed as such, is easily dispersed in water. The assessment presented here is nested in the CATALYST study, a phase I/II pharmacokinetic, safety, and acceptability trial.^15^

## METHODS

**Figure 1.**
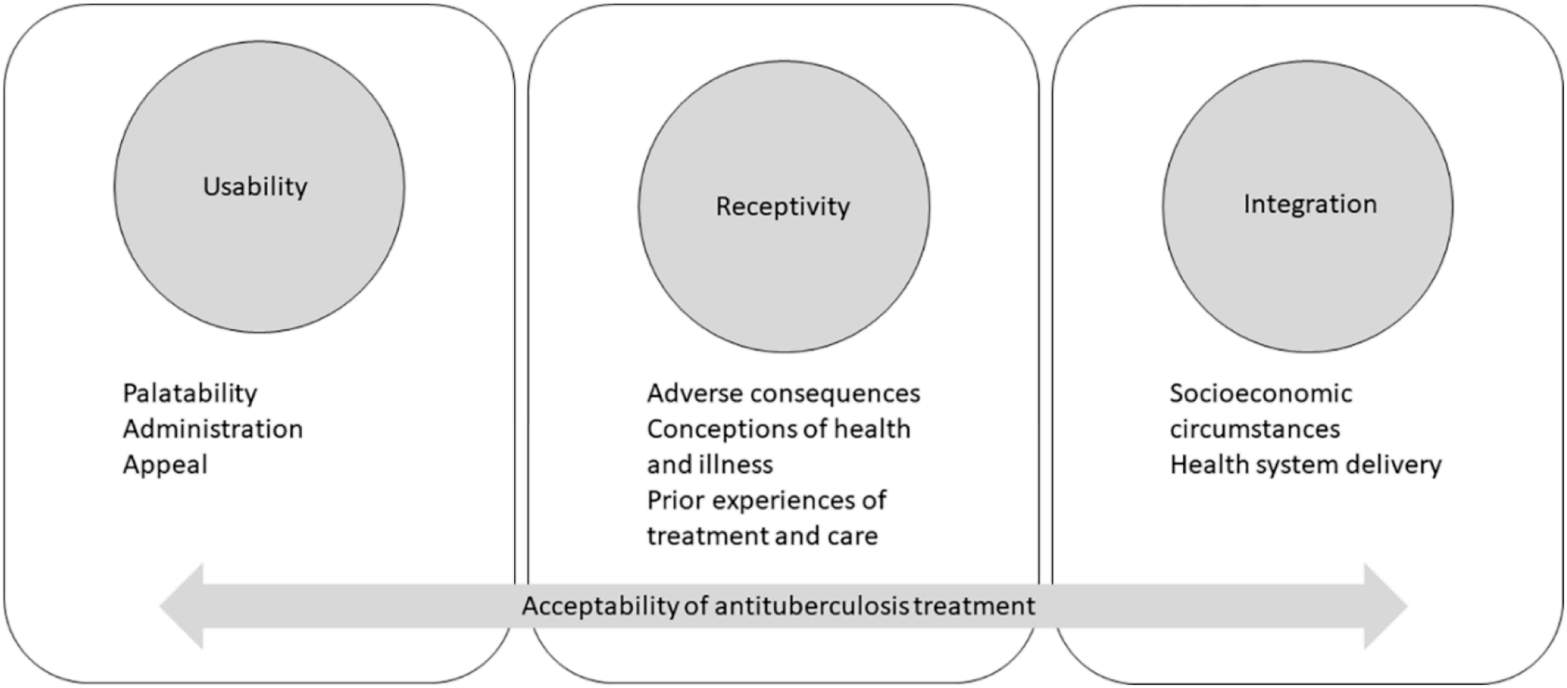
Wademan et al.^12^ conceptual framework for TB treatment acceptability.

We conducted a comparative case description study. Drawing on the multi-domain Wademan TB treatment acceptability model (Figure 1),^12^ we describe the usability and receptivity domains of the CFZ tablet for child-caregiver dyads. We did not engage with the ‘integration’ component as the acceptability assessment was conducted in a trial setting. For the trial, participants also received a novel dispersible moxifloxacin tablet (data reported elsewhere) alongside other drugs that are part of their routine treatment regimen.

### Setting and study design

The CATALYST trial, including the nested acceptability evaluation, was conducted between March 2021 to July 2023. Data were collected at a specialised TB hospital in Cape Town, South Africa; a large tertiary private hospital in Region IV-A (Calabarzon), Luzon, Philippines; and in Pune, India at a Government Medical College.

**Figure 2.**
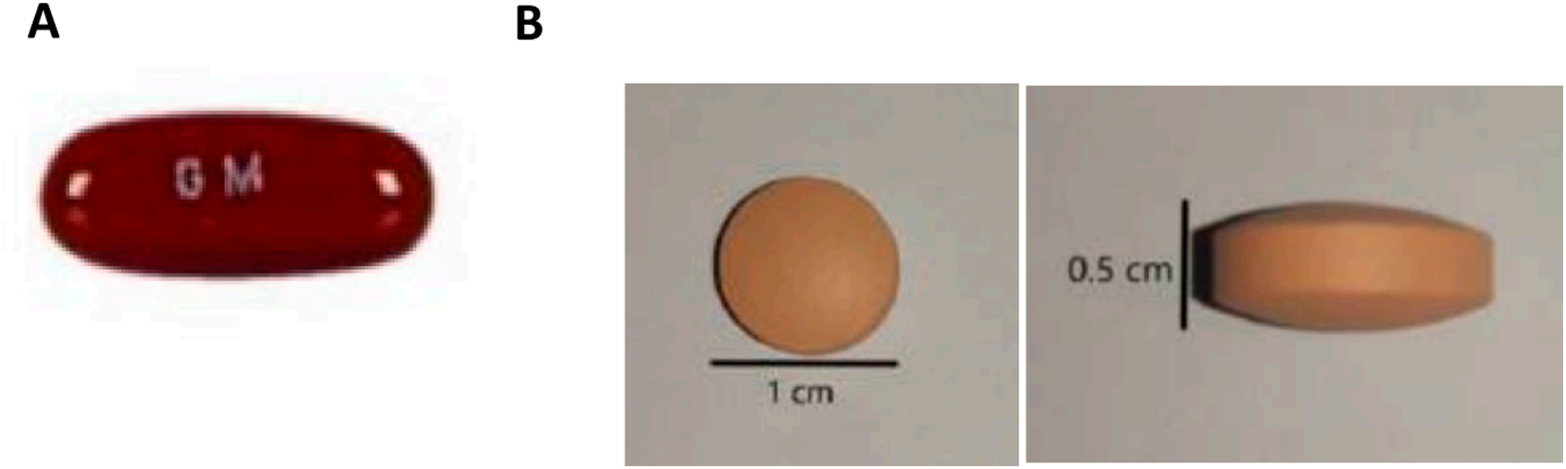
**A**: Routine clofazimine 100mg gel capsules. **B**: the novel 50mg clofazimine tablet.

Children were eligible for the CATALYST trial if they were 0–14 years-old, routinely diagnosed with RR-TB, and living with or without HIV. All children enrolled in the CATALYST trial (or their caregivers as proxies) completed quantitative acceptability questionnaires and a sub-sample participated in qualitative interviews. At enrolment, all children were on CFZ, receiving either the 100mg or 50mg gel capsule, or a tablet formulation, depending on availability of treatment in routine care (Figure 2). Children taking the standard 100mg gel capsule (‘the gel capsule’) and caregivers were instructed to administer this whole. Children then switched to the novel 50mg CFZ tablet formulation (‘the clofazimine tablet’) and caregivers were instructed to disperse this formulation in water for administration. This method of administration was important for the two days preceding the pharmacokinetic study visits but outside of these days the CFZ tablet could be administered as preferred (swallowed whole, chewed, dispersed in water, or mixed with food or drinks).

### Qualitative sub-sample and recruitment

A purposive sub-sample of child-caregiver dyads were selected for diversity in country, sex, and age, and to saturation of meaning (i.e., the point when an issue is fully understood, and when no further dimensions, nuances, or insights emerge).^16^ These participants were recruited by the clinical trial staff asking them/their caregivers if they were interested in sharing their experiences of taking RR-TB treatment and then completing informed consent.

### Data collection

The quantitative data were collected through questionnaires focusing on treatment experiences and administration practices for both the gel capsule and the CFZ tablets as well as challenges of ingestion and administration. Participants completed questionnaires at Week 0 (study enrolment), Week 2 (after routine gel CFZ formulation had been switched to the novel CFZ tablet formulation), Week 8 and Week 24 (study completion) – see Supplementary Data. These were administered by the study team using electronic devices to the child or, if they were too young/unable, their caregiver as proxy, with responses entered directly into a REDCap database.^17^ The questionnaire was available in English, translated into relevant local languages (Afrikaans or Xhosa in South Africa; Marathi or Hindi in India, and Tagalog in the Philippines) and was piloted to ensure fidelity among participants. The data collectors were multi-lingual and used structured and consistent questioning approaches when interacting with the participants as outlined during training.

The qualitative data were collected through in-depth interviews (∼45 minutes each) conducted over ∼4 interactions over the child’s 24-week trial enrolment (Week 2, 4, 16, 24) with the child and/or caregiver. Three of the interviews were conducted at the trial site, commencing shortly after the participant switched from CFZ gel capsule to tablet formulation. The fourth was planned as a home visit, where possible and where participants agreed, although this was often not feasible due to COVID-19 pandemic restrictions, or in some cases, stigma and fear of disclosure.^18^ The interviews included participatory activities such as kinship maps, timelines, and body mapping and followed a semi-structured discussion (see Supplementary Data). Qualitative data were collected by trained female postgraduate researchers (LV, NTCC, NS, AM, AMMC, JDDO, GM, and SB) fluent in participants’ preferred language (as above) and audio recorded. Researchers wrote detailed case narratives, including verbatim quotations and interviewer reflections after each discussion.^19^ Regular bi-weekly iterative analytic debriefs were done within and across country partners to ensure data consistency.

### Data analysis

The unit of analysis for this study is the child-caregiver dyad. Age and sex were summarized in aggregate and by country site using descriptive statistics. Summary statistics were used to describe administration experiences related to (1) how routine gel capsules and novel CFZ tablet formulations were administered or (2) mixed with anything to either make the medicine easier to swallow or to hide the taste/make it taste better were described. The Likert acceptability data were described using frequencies and percentages at Week 0, Week 2, Week 8 and Week 24 and were dichotomized as follows: Strongly Agree/Agree/Neutral vs Disagree/Strongly Disagree. The routine gel capsule CFZ at Week 0 and the novel CFZ tablet at Week 2 were compared using McNemar test. All quantitative data were analysed using Stata Special Edition 16.1 software.^20^ Qualitative data analysis was iterative and followed a deductive thematic approach informed by the Wademan acceptability framework. Cross-country case descriptions were combined into a matrix by co-authors and cross-checked during a workshop led by the first author. Data are reported using the COREQ checklist (Supplementary Data).

### Ethics

The trial, including the nested acceptability evaluation, was approved by the Health Research Ethics Committee at Stellenbosch University, the South African Health Products Regulatory Authority (SAHPRA), BJGMC Ethics Committee in India, the De La Salle University of Research Ethics Office (DLSU-REO) in the Philippines, and by the WHO Research Ethics Review Committee. Informed consent was provided by the participants’ parents or guardian. Children ≥7-years-old also provided assent. Consent and assent for the qualitative component were provided separately without bearing on overall trial participation.

## RESULTS

The quantitative questionnaires were administered to 36 children/caregivers. The median age of child participants was 4.9 years and the mean age at enrolment was 4.1 years in South Africa, 7.7 years in India, and 8.1 years in the Philippines. The qualitative interviews were conducted with a subset of 26 child-caregiver dyads, with all participants enrolled in India participating in the interviews (Table 1).

**Table 1.** Demographics of CATALYST population: participants completing questionnaires and participating in interviews.

**Questionnaires**				
	Total (n=36)	South Africa (n=20)	India (n=6)	Philippines (n=10)
Sex				
Male (%)	14 (38.9)	9 (45.0)	1 (16.7)	4 (40.0)
Female (%)	22 (61.1)	11 (55.0)	5 (83.3)	6 (60.0)
Age				
0-7 years old	27 (75.0)	19 (95.0)	3 (50.0)	5 (50.0)
8-14 years old	9 (25.0)	1 (5.0)	3 (50.0)	5 (50.0)
**Qualitative interviews**				
	Total (n=26)	South Africa (n=13)	India (n=6)	Philippines (n=7)
Sex				
Male	11	7	1	3
Female	15	6	5	4
Age				
0–7 years old	19	12	3	4
8–14 years old	7	1	3	3

### Usability

In total, 29/36 (80.6%) children reported having ever taken the gel capsule CFZ formulation and 17 (58.6%) reported swallowing the capsule whole. Outside of the days preceding the pharmacokinetic visits, where participants were advised to disperse the CFZ tablet, 9/36 participants (25.0%) swallowed the CFZ tablet whole without dispersing (Table 2). Table 3 displays the frequencies and percentages of ingestion facilitation agents/flavourings participants mixed with CFZ gel capsules and CFZ tablets. Over 70% of participants reported using water to facilitate administration (77.8%; CFZ gel capsules: 21/29, 72.4%; CFZ tablets: 33/36, 91.7%)

**Table 2.** Administration of the clofazimine (CFZ) gel capsule and the novel CFZ tablet formulation.

	CFZ gel capsule (Week 0)	Novel CFZ tablet (Week 2)
	(n=29)*	(n=36)
**How the CFZ gel capsules are administered.**		
Swallowed whole (%)	17 (58.6)	
Chewed (%)	2 (6.9)	
Softened in liquid/food (%)	5 (17.2)	
Extracting from capsule with a syringe and drinking the liquid (%)	4 (13.8)	
Extracting from capsule and mixing with food (%)	1 (3.4)	
**How the novel CFZ tablets are administered.**		
Swallowed whole (%)		9 (25.0)
Chewed (%)		0 (0.0)
Dispersed in water (%)		27 (75.0)

* Participants receiving gel capsules as part of routine care.

**Table 3.** Agents used to facilitative swallowing or improve taste of clofazimine (CFZ) gel capsules vs CFZ tablet formulations.

	Mixed to make it easier to swallow^A^		Mixed to hide the taste or to make it taste better^A^	
	CFZ gel capsules Week 0	CFZ tablets Week 2	CFZ gel capsules Week 0	CFZ tablets Week 2
	(n=29)	(n=36)	(n=29)	(n=36)
Nothing (%)	6 (20.7)	2 (5.6)	12 (41.4)	11 (30.6)
Water (%)	21 (72.4)	33 (91.7)	9 (31.0)	21 (58.3)
Yoghurt (%)	2 (6.9)	1 (2.8)	5 (17.2)	2 (5.6)
Porridge or ‘pap’ (%)	0	0	0	0
Milk (%)	0	0	0	0
Tea or Coffee (%)	0	0	0	1 (2.8)
Juice (%)	0	0	0	1 (2.8)
Banana (%)	0	0	0	0
Honey (%)	0	2 (5.6)	1 (3.4)	2 (5.6)
Sugar (%)	3 (10.3)	1 (2.8)	5 (17.2)	3 (8.3)
Other (%)	1 (3.4)^B^	1 (2.8)^C^	1 (3.4)^D^	3 (8.3)^E^

A Multiple options possible;

B Chocolate;

C Buko shake;

D Chocolate drink, peanut butter and chocolate;

E Buko shake, jaggery, sweet dish.

Qualitatively, participants described some of the challenges of administering the gel capsule including the difficult process of softening the capsule or extracting the liquid for administration, especially to younger children. For instance, the mother of a girl (3 y/o, Philippines) described that even softening the capsule in water would not help with administration and she would have to pierce the capsule and empty the contents into a cup to administer the whole dose. Similarly, the mother of a boy (2 y/o) in South Africa explained:

“I used to give [my son] a red capsule [100mg gel capsule clofazimine], but it was hard to open [to] take out the liquid that was inside the capsule.”

For the CFZ tablet formulation, most participants opted to disperse the tablet in water, describing it as easy to dissolve. The same mother in South Africa said:

“the new [tablet] treatment dissolves quickly in water, like Aspirin. The new treatment is better because he can just take it with water.”

These findings were consistent across settings, with little variation in terms of context-specific challenges in treatment administration challenges. Although administering the CFZ tablet was described as substantially better than the gel capsule, participants still experienced challenges with administering multiple drugs and often multiple pills per drug every day. Caregivers described treatment administration as a lengthy process, involving breaks, ‘bribes’, or support. The improved ease of preparation and ingestion of the CFZ tablet was important but marginal in the context of regimens that are generally difficult to prepare and administer. For children who received 100mg tablets as part of routine care prior to switching to the novel 50mg CFZ tablet formulation, caregivers also described how the novel formulation was an improvement. One mother in India described how previously she had to cut the CFZ tablet that was prescribed in routine care into halves and, after administering the one half to her child, had to store the other half in a secure place for subsequent administration. With the novel 50mg tablet, she administered the whole dose, saving time and easing administration burden (Mother of girl, 8 y/o, India). Participants also explained how they incorporated either food or drinks into their administration process. The father of a boy (7y/o, Philippines) described their daily treatment routine:

“[I] usually administer [the boy’s] tablets whole for him to swallow […] but it became routine [and we have] learned what techniques work […]. Sometimes [he] eats the food with the medicine or drinks the juice […]. When he’s done … he would eat the rest of the food as a sort of ‘chaser.’”

The caregiver of a girl (7 y/o, India) related the lengthy process of administering 8–9 tablets for RR-TB treatment. The girl would swallow the CFZ tablet whole, followed by some water. She explained that mixing all dispersible medications was one way to ease the burden of administration:

“It is always better to mix them once with water than to swallow them three times.”

Overall, in the quantitative data, participants had favourable responses to the size of the CFZ, both for the gel capsule and the tablet. However, participants described how the child would be upset when receiving the gel capsule CFZ than when receiving the CFZ tablet formulations (Table 4), although this could potentially be attributed to improved health and becoming used to receiving medication.

**Table 4.** Acceptability of clofazimine (CFZ) gel capsule at Week 0 and CFZ tablet at Week 2, Week 8 and Week 24.

	CFZ gel capsules Week 0	CFZ tablet Week 2	CFZ tablet Week 8	CFZ tablet Week 24
	(n=29)	(n=36)	(n=35)	(n=33)
The size of Clofazimine makes it easy for my child/me to swallow.				
Strongly agree, agree or neutral (%)	22 (75.9)	35 (97.2)	31 (88.6)	28 (84.8)
Disagree or strongly disagree (%)	7 (24.1)	1 (2.8)	4 (11.4)	5 (15.2)
(My child gets/ I get) the whole dose of Clofazimine every time it is administered.				
Strongly agree, agree or neutral (%)	29 (100.0)	36 (100.0)	35 (100.0)	33 (100.0)
Disagree or strongly disagree (%)	0	0	0	0
(My child is/ I am) upset every time Clofazimine is administered.				
Strongly agree, agree or neutral (%)	12 (41.4)	8 (22.2)	7 (20.0)	6 (18.2)
Disagree or strongly disagree (%)	17 (58.6)	28 (77.8)	28 (80.0)	27 (81.8)
(My child likes/I like) the taste of Clofazimine.				
Strongly agree, agree or neutral (%)	16 (55.2)	29 (80.6)	31 (88.6)	28 (84.8)
Disagree or strongly disagree (%)	13 (44.8)	7 (19.4)	4 (11.4)	5 (15.2)
(My child thinks/I think) the smell of Clofazimine is gross/yucky.				
Strongly agree, agree or neutral (%)	17 (58.6)	15 (41.7)	9 (25.7)	7 (21.2)
Disagree or strongly disagree (%)	12 (41.4)	21 (58.3)	26 (74.3)	26 (78.8)
I believe the Clofazimine is improving (my child’s/my) health.				
Strongly agree, agree or neutral (%)	27 (93.1)	36 (100.0)	34 (97.1)	33 (100.0)
Disagree or strongly disagree (%)	2 (6.9)	0	0	0
The side effects that (my child has experienced/I experienced) are unbearable.				
Strongly agree, agree or neutral (%)	7 (24.1)	2 (5.6)	4 (11.4)	3 (9.1)
Disagree or strongly disagree (%)	22 (75.9)	34 (94.4)	31 (88.6)	30 (90.9)

### Palatability

Table 3 displays the frequencies and percentages of ingestion flavourings participants mixed with CFZ gel capsules and CFZ tablets. Some participants reported using various agents to mask the taste of CFZ. Between 30-60% used water to improve taste (CFZ gel capsules: 9/29, 31.0%; CFZ tablets: 21/36, 58.3%). Sweetening agents such as yoghurt, honey and sugar were used by fewer than 15% of participants. Participants generally had more positive responses to the palatability, including smell, taste, aftertaste, and mouth feel, of the CFZ tablet, especially compared to the gel capsule. More participants reported favourably (strongly agree/agree/neutral) that they liked the taste of CFZ tablet than the CFZ gel capsules. Similarly, more participants agreed or were neutral the smell of CFZ gel capsule was gross or yucky when compared to the smell of the novel CFZ tablets – see Table 4. In the qualitative data, the smell of the gel, if extracted from the 100mg gel capsule formulation, was described as strong, lingering, and nauseating. The caregiver of a 7 y/o, boy from South Africa described how:

“When he started with treatment, there was a brown [capsule], clofazimine, which [he] did not like. He was saying it smelled like powder soap. He would quickly drink the [medicine] then hold his breath, so he does not smell it. [He] said when he would burp [belch], he would smell clofazimine on his breath.”

Other participants noted that the smell itself was not the challenge (comparing it to chocolate), but the smell along with the associated taste led to a strong aversion:

“If I smell that, I don’t want [to take it] anymore […] It smelled like chocolate at first, then you taste it and […] you don’t like it anymore” (13y/o girl, Philippines).

When describing the 50mg CFZ tablet, the same participant noted:

“I was very happy when I found out [that the clofazimine tablet has] no taste, no smell… It's easy to drink. I can just chew on fruits or biscuits that have some taste. I don’t need to drink [chocolate milk].”

Positive experiences with the palatability of the novel 50mg tablet formulation were reported across settings, regardless of differences in how the medicine was taken and potentially culturally specific preferences for taste and/or smell.

### Appeal

Generally, participants had no overwhelming responses to the appeal of the 50mg CFZ tablet. When dispersed/crushed in water, the tablet was orange in colour with a slightly grainy texture. One participant from the Philippines found this particularly unappealing – although this was an outlying experience. The 14 y/o girl noted that just seeing the colour of the dispersed CFZ tablet as it was being prepared made her “want to vomit”. However, she still described this as an improvement on existing gel capsules.

### Receptivity – perceptions of health

Participants held strong beliefs that CFZ, both the gel capsule and the tablet formulation, would be able to improve the child’s health, with all participants agreeing/strongly agreeing at all time points with the statement. Only 2/29 (6.9%) participants who had ever taken the gel capsule and 1/35 (2.9%) participant taking CFZ tablet at Week 8 reported their belief that CFZ was not improving the child’s health. More participants, 7/29 (24.1%) reported that the side effects of the treatment were unbearable at Week 0 (gel capsules), compared to the CFZ tablets at Week 2 (2/36 5.6%), Week 8 (4/35; 11.4%), and Week 24 (3/33; 9.1%) – Table 4.

### Adverse consequences

Although participants had overall positive responses to the novel tablet formulation, both the 100mg gel capsule and 50mg tablets were noted to stain clothing and furniture. The caregiver of a 5 y/o boy in South Africa showed the researchers a stained cloth and explained that the CFZ:

“stained [the boy’s] clothes and blankets when he would spit-up or vomit.”

However, the 50mg tablet was easier to manage because it could be dispersed in a cup/container with less accidental contact with other surfaces. Participants also noted skin changes, including hyperpigmentation, which several caregivers and children described as upsetting. Changes in skin colour meant that participants’ TB diagnosis could inadvertently be disclosed to others, which could lead to stigma.^21^ For instance, the caregiver of one boy (11 y/o, India) described how the boy’s skin got darker:

“[we] haven’t experienced discrimination due to TB before [but the hyperpigmentation] was leading to gossip.”

### Comparative experiences: gel capsule vs tablet formulations

When comparing the tablet formulation to the routine gel capsule, participants reported significant differences in terms of ability to swallow and observed side effects, with higher positive scores for the 50mg tablet - Table 5. There was a difference of -20.7% (95% CI: -41.7, 0.0, p-value=0.034) in the disagreement that the size of CFZ made it easier for the child to swallow for the gel capsule to novel CFZ tablet (24.1% to 3.5%). There was a 17.2% difference (95% CI: -3.0, 37.4, p-value=0.059) in the disagreement that the side effects were unbearable for the gel capsule to CFZ tablet (75.9% to 93.1%). There was a 17.2% difference (95% CI: -3.0, 37.4, p-value=0.059) in the disagreement that the side effects were unbearable for the gel capsule to CFZ tablet (75.9–93.1%).

**Table 5.** Comparison acceptability of clofazimine (CFZ) gel capsule and CFZ tablet at Week 2 (n=29).

	CFZ gel capsule Strongly disagree or disagree (%)	CFZ tablet Strongly disagree or disagree (%)	% Difference (95 % CI)	p-value
My child likes the taste of Clofazimine.	13 (44.8)	7 (24.1)	-20.7 (-44.1, 2.8)	0.058
My child thinks the smell of Clofazimine is gross/yucky.	12 (41.4)	16 (55.2)	13.8 (-8.1, 35.7)	0.157
The size of Clofazimine makes it easy for my child to swallow.	7 (24.1)	1 (3.5)	-20.7 (-41.7, 0.0)	0.034
My child gets the whole dose of Clofazimine every time it is administered.	0	0	(-0.03, 0.03)	1.000
My child is upset every time Clofazimine is administered.	17 (58.6)	21 (72.4)	13.8 (-10.4, 38.0)	0.206
I believe the Clofazimine is improving (my child’s/my) health.	2 (6.9)	0	-6.9 (-19.6, 5.8)	0.157
The side effects that (my child has experienced/I experienced) are unbearable.	22 (75.9)	27 (93.1)	17.2 (-3.0, 37.4)	0.059

## DISCUSSION

The novel 50mg CFZ tablet was generally well received by children treated for RR-TB and their caregivers. For usability, the tablet was, overall, easier to prepare and administer, more palatable, and more appealing compared to the current standard 100mg gel capsule. Participants noted that the novel formulation had a neutral taste and smell, especially compared to the standard formulation that was described as foul-smelling with a lingering odour. Although the novel tablet formulation was seen as an improvement, there were still challenges with appeal as CFZ had an unappealing colour when dissolved. For receptivity, staining continues to present a challenge, especially to caregivers of young children. Hyperpigmentation was also concerning to participants, which often led to unanticipated disclosure of their TB diagnosis. Participants trusted in CFZ, both the gel capsule and the tablet formulation, and that the children’s health would improve.

Strengths of our analysis included the mixed-method approach allowing for triangulation, collecting data longitudinally and in three diverse high burden settings, using participatory activities to include children’s voice directly, and the fact that these are the first data reporting on acceptability of this novel CFZ tablet formulation. Limitations to generalizability are that the sample size for the quantitative data is small and skewed toward participants from South Africa and that these were children enrolled in a clinical trial, which is a different experience to receiving care through routine health care services only. Participants’ perceptions of the side effects or palatability of the novel CFZ tablet is potentially influenced by their experience of taking multiple other drugs as part of their RR-TB treatment regimen, although the in-depth qualitative research methods were designed to mitigate some of these challenges. Additionally, integration as a domain of acceptability was not explored as this analysis was conducted in a trial setting.

A previous single-site study in South Africa has shown that a 50mg gel capsule is comparatively palatable and acceptable compared to a 100mg gel capsule, but that children and caregivers still experienced challenges with treatment administration.^22^ In a review for the WHO, researchers conducted a retrospective qualitative evaluation of the paediatric age-appropriateness of formulations on the List of Essential Medicines for Children (EMLc). They found that CFZ in capsule form (both 50mg and 100mg) had lower acceptability and palatability scores when considered for administration in younger children compared to older children, emphasising the need to consider the age-specific needs and preferences of children.^23^ Gel capsule formulations of drugs continue to present a challenge when dosing requires administering portions of the encapsulated gel or when administered in conjunction with multiple other medications.^22^ Solid tablet formulations are generally more child-friendly, as they are easier to split, crush, or dissolve or swallow whole.^24,25^ A recent study found that weight-adjustable, mini-tablets of CFZ are potentially more suitable for administration in children as young as 6 months of age.^26^

Given its much improved acceptability, the 50mg tablet formulation should be prioritised for children with RR-TB over the currently available 100mg gel capsule. Commonly reported side effects of CFZ in the literature, including diarrhoea, vomiting, and abdominal pain and the inconvenience of skin ‘staining’ will not, however, be addressed by changing the formulation and these remain challenges to the overall acceptability of this drug for children with RR-TB.^6^ Similarly, the challenging social circumstances that many children with RR-TB experience will often overshadow any incremental improvement to the formulation.^27,28^ Future research should explore how best to integrate innovations in RR-TB care for children into regimens, dose preparation, and administration and adherence support tools that can be scaled for use in routine health care settings.

## Supplementary Material


