# Supplementary material for: Acceptability of a clofazimine tablet in children with rifampicin-resistant TB in three high-burden countries
Source: IJTLD Open. 2025 Aug 13;2(8):478–85. doi: 10.5588/ijtldopen.25.0309 (PMC12352948; doi:10.5588/ijtldopen.25.0309)
Supplement: Supplementary file 1 [file ijtldopen25-0309_supplementarydata1.pdf]

Supplementary File 1: Quantitative Questionnaire used to assess acceptability in the CATALYST Trial.

S3_IGN	Section 3. Past experiences with Clofazimine ingestion/administration **[WEEK 0]**		
S3_Inst	I am now going to ask you a few questions about you/your child's general experiences with the (100mg gel capsule) Clofazimine formulation		
S3_Taken	Have you/r child ever taken those clofazimine 100mg gel capsule (refer to image)?	1, Yes<br>2, No	3, I don't know
S3_Admin	How is/was the 100mg gel capsule formulation Clofazimine capsules usually administer to you/the child?	1, Swallowed whole<br>2, Chewed<br>3, Softened in liquid/food	4, Extracting from capsule with a syringe and drinking the liquid<br>5, Extracting from capsule and mixing with food
S3_MixSwall	Which of the following is/was usually mixed with the capsules *to make it easier for you/them to swallow/take all of the dose?* Please indicate all that apply.	1, Nothing<br>2, Water<br>3, Yoghurt<br>4, Porridge/pap<br>5, Milk<br>6, Tea/Coffee	7, Juice<br>8. Banana<br>9. Honey<br>10. Sugar<br>99, Other (specify)
S3_MixTaste	Which of the following is/was usually mixed with the capsules to *hide the taste of the medicine or make it taste better for the child?* Please indicate all that apply.	1, Nothing<br>2, Water<br>3, Yoghurt<br>4, Porridge/pap<br>5, Milk<br>6, Tea/Coffee	7, Juice<br>8. Banana<br>9. Honey<br>10. Sugar<br>99, Other (specify)
S3_Inst2	I am now going to read you a series of statements about the 100mg gel capsule formulation **Clofazimine** (refer to picture) For each statement I want you to answer either: "Strongly agree", "Agree", "Neutral", "Disagree", or "Strongly disagree". Remember I am asking about the 100mg gel capsule Clofazimine formulation, not the new one from the study. (Show participant image of scale labelled [Strongly agree - Strongly disagree] at the beginning of the section and again as needed.)		
S3_Taste	I/My child really *likes the taste* of 100mg gel capsule formulation Clofazimine capsules.	1, Strongly agree<br>2, Agree<br>3, Neutral	4, Disagree<br>5, Strongly disagree
S3_Smell	I/My child thinks that the *smell* of 100mg gel capsule formulation Clofazimine. capsules is *gross/yucky*.	1, Strongly agree<br>2, Agree<br>3, Neutral	4, Disagree<br>5, Strongly disagree

S3_Size	The *size* of 100mg Clofazimine gel capsule makes it *easy* for my child *to swallow*.	1, Strongly agree<br>2, Agree<br>3, Neutral	4, Disagree<br>5, Strongly disagree
S3_WholeDos	I am sure that I/my child gets the *whole dose* (as prescribed by the doctor) of the 100mg Clofazimine gel capsule every time that it is administered.	1, Strongly agree<br>2, Agree<br>3, Neutral	4, Disagree<br>5, Strongly disagree
S3_Tension	*Administering/taking* the 100mg Clofazimine gel capsule has become a source of *unhappiness or tension* in my family.	1, Strongly agree<br>2, Agree<br>3, Neutral	4, Disagree<br>5, Strongly disagree
S3_Upset	My child/I am/is *upset* every time the 100mg Clofazimine gel capsule is administered.	1, Strongly agree<br>2, Agree<br>3, Neutral	4, Disagree<br>5, Strongly disagree
S3_ImprHea	I believe that the 100mg Clofazimine gel capsule are *improving my/my child's health*.	1, Strongly agree<br>2, Agree<br>3, Neutral	4, Disagree<br>5, Strongly disagree
S3_SideEff	The *side effects* that I/my child has experienced from the 100mg Clofazimine gel capsule are *unbearable*.	1, Strongly agree<br>2, Agree<br>3, Neutral	4, Disagree<br>5, Strongly disagree
S4_ING	Section 4. Experiences with novel Clofazimine formulation ingestion/administration **[WEEK 2, 8, 24]**		
	I am now going to ask you a few questions about you/your child's general experiences with the novel Clofazimine 50mg tablet formulation. These are the small, round brown/orange tablets (Show participant picture of novel formulation Clofazimine)		
S4_Admin	How is the (50mg) round brown/orange Clofazimine tablets usually administered to the child?	1, Swallowed whole<br>2, Chewed	3, Dispersed in water<br>4, Other (specify)
S4_MixSwall	Which of the following is/was usually mixed with the round tablets with to make it easier for them to swallow/take all of the dose? Please indicate all that apply.	1, Nothing<br>2, Water<br>3, Yoghurt<br>4, Porridge/pap<br>5, Milk<br>6, Tea/Coffee	7, Juice<br>8, Banana<br>9, Honey,<br>10, Sugar<br>99, Other (specify)
S4_MixTaste	Which of the following is/was usually mixed with the (50mg) round brown/orange Clofazimine tablets with to hide the taste of the medicine or make it taste better for you/the child? Please indicate all that apply.	1, Nothing<br>2, Water<br>3, Yoghurt<br>4, Porridge/pap	7, Juice<br>8, banana<br>9. Honey<br>10, sugar

		5, Milk<br>6, Tea/Coffee	99, Other (specify)
S4_Inst2	I am now going to read you a series of statements about the new 50mg Clofazimine formulation (the small, round brown/orange tablets). (Show participant picture of new formulation Clofazimine) For each statement I want you to answer either: "Strongly agree", "Agree", "Neutral", "Disagree", or "Strongly disagree". (Show participant image of scale labelled [Strongly agree - Strongly disagree] at the beginning of the section and again as needed.)		
S4_Taste	I/My child really likes the taste of the (50mg) round brown/orange Clofazimine tablets.	1, Strongly agree<br>2, Agree<br>3, Neutral	4, Disagree<br>5, Strongly disagree
S4_Smell	I/My child thinks that the smell of the (50mg) round brown/orange Clofazimine tablets is gross/yucky.	1, Strongly agree<br>2, Agree<br>3, Neutral	4, Disagree<br>5, Strongly disagree
S4_Size	The size of the (50mg) round brown/orange Clofazimine tablets makes it easy for me/my child to swallow.	1, Strongly agree<br>2, Agree<br>3, Neutral	4, Disagree<br>5, Strongly disagree
S4_WholeDos	I am sure that I/my child gets the whole dose (as prescribed by the doctor) of the (50mg) round brown/orange Clofazimine tablets every time that it is administered.	1, Strongly agree<br>2, Agree<br>3, Neutral	4, Disagree<br>5, Strongly disagree
S4_Tension	Administering the (50mg) round brown/orange Clofazimine tablets has become a source of unhappiness or tension in my family.	1, Strongly agree<br>2, Agree<br>3, Neutral	4, Disagree<br>5, Strongly disagree
S4_Upset	I/My child am/is upset every time (50mg) round brown/orange Clofazimine tablets are administered	1, Strongly agree<br>2, Agree<br>3, Neutral	4, Disagree<br>5, Strongly disagree
S4_ImprHea	I believe that the small round brown/orange tablets are improving my child's health.	1, Strongly agree<br>2, Agree<br>3, Neutral	4, Disagree<br>5, Strongly disagree
S4_SideEff	The side effects that I/my child has experienced from the (50mg) round brown/orange Clofazimine tablets are unbearable.	1, Strongly agree<br>2, Agree<br>3, Neutral	4, Disagree<br>5, Strongly disagree

Supplementary File 2: Discussion guide used to assess acceptability in the CATALYST Trial.

**Discussion guide:** ***Clofazimine and moxifloxacin PK, safety, and AccepTAbiLitY for paediatric TB treatment. (CATALYST)***

<u>Purpose:</u> This document is to be completed by the research staff completing discussions with key informants who form part of the CATALYST study. The aim of the study is to understand children receiving Rifampicin resistant TB (RR-TB) treatment and their caregivers' perceptions of treatment palatability and acceptability and to understand how their experiences develop over the course of the treatment journeys. The discussions should be completed with either the caregiver or the child participant, depending on the age of the participant and their ability to participate.
<u>Note:</u> The activities and questions in this discussion guide are expected to be completed in <u>3-4</u> interviews. The first of the interviews will likely coincide with their enrolment visit. The remaining discussions/topic areas will be conducted at <u>approximately</u> 4, 16, and 24 weeks after enrolment.
<u>Expected time needed per use:</u> 45~60 minutes per interview.

<u>Visit 1: Week 0 (facility)</u>

<u>Kinship map activity</u>

Aim: To draw a picture that represents (1) the participants' "family" (biological and non-biological), (2) the types of relationships between each member of that "family", and (3) cohabitation/co-residence of people ("family" and non-family) with the participant.

1. Researcher to tell the participant: "*We are going to be doing a Kinship Map with you today. A kinship map is like a family tree, or something we use to draw your family and household. It will help us to see who you live with and help us to get to know you and your family. We are going to draw the family tree or kinship map together*." If the child is old enough, they can participate with the help of the caregiver/adult.
2. Ask the participant to create a list of people that are part of their <u>family</u> and <u>household</u>.
3. Ask about co-residence/co-habitation.

   Additional probes:
1. Please indicate, which members of the family are aware of the child's diagnosis?
2. Please indicate which family members have ever been affected by TB or RR-TB?

<u>Household floorplan activity</u>

Aim: To 1) draw the infrastructure of the participant's house and spaces that they use on a daily basis; 2) understand the participant's perception of their home and how they relate to their house/home; 3) understand the participant's perception of how other people experience their house/living space and, overall, how it relates to illness management. The participant should either be the child if they are able (with assistance from the caregiver where needed) or the adult caregiver, as appropriate.

1) Researcher to participant: "*As part of this activity we will be drawing a floorplan for your house to better understand the layout of your home and how different people use the space. This will help us understand if the ways in which households influence the ways in which health or illnesses are managed*."
2) Draw an outline of their house and the yard overall; draw the inner lines of the house; draw the furniture; explain how different spaces are used/shared

<u>Visit 2: Week 4 (facility)</u>

<u>Illness narrative and timeline activity</u>

Aim: To understand the illness journey of the participant from the beginning (pre-diagnosis through diagnosis and treatment) and how the journey evolved overtime.

1. "*We are going to be doing an illness journey timeline activity. An illness journey allows researchers and health workers to understand your (or your child's) unique experiences of living with TB. This also allows researchers and health workers to understand the role TB plays in your life and the*

impact that TB has had on it. The timeline will allow us to see the 'main/important events' that has occurred in your/your child's life."

2. Ask the participant to tell you the story of how they (child patient) managed to become enrolled in this study. Plot the points such as diagnosis, symptoms, referrals, and major events next to their age.
3. Ask the participant to walk you through their journey of when and at what age they had their first experience/encounter with TB and ask them to note this on the page/timeline.

Illness narrative and timeline follow-up questions: Treatment (Repeated at Visit 3 and 4)

Aim: Building on the illness narrative/timeline, to explore the ways in medications are viewed and experienced by child patients (or their caregivers). This includes exploring 1) the ways in which the medications are administered; 2) overall perceptions of the medications; 3) overall acceptability/palatability of the medications.

1. Explain to participant: "*We have discussed your treatment and illness journey up to now. We can now talk about the new treatment that you are currently taking."*
2. Please describe all the medication you/child are taking as part of the study treatment and how they are taken (packaging, taste, feel, etc.)
3. What have been the difficult things about taking/giving these treatments?

Body map activity

Aim: To draw a picture that represents (1) the participants' body; (2) to explore how the participant perceives their body; 3) to understand how the participants experience their health condition(s); and 4) to explore the participant's emotional-physical-social experience as a result of their health condition(s), including the treatment they receive.

1) "*We would like to understand how RR-TB has affected your body. To do that, we are going to draw a 'body map'. A body map is a life-sized (or printed) outline of your body where you can fill in or draw details*."
2) Ask the participant to mark all the places on their body where TB affects them/their child.
3) Ask the participant to expand on how the treatment makes their body feel.

Visit 3: Week 16 (facility)

Illness narrative follow-up questions: Treatment (Repeat activity – as per visit 2)

Health beliefs Action activity

Aim: The aim of this activity is 1) to understand the thought process that influences health seeking behaviours in participants; 2) to understand how perceptions of susceptibility and severity of disease influence choices to prevent disease and adhere to treatment; and 3) to understand how individual's perceptions of how they SHOULD behave, influences their eventual decisions to either prevent a disease and/or adhering to treatment. Either the child or the caregiver should complete the activity, depending on the age/capabilities of the child.

1) Tell the participant" "*As part of this next activity, we want to find out what are the things that influence how people think about getting health care, how people understand illnesses, including how bad illness can be or how likely they are to become ill, and what people think about what people are and should be doing when taking treatment.*"
2) Areas to explore:
    a. Susceptibility
    b. Severity
    c. Control over outcomes (general)
    d. Ability to implement behaviour (specific)
    e. Descriptive normative expectations
    f. Injunctive normative expectations

Health beliefs

Aim: To understand how participant makes sense of and manage their health. By discussing the different health systems that participants engage with, we are better able to understand how participants make decisions around accessing care or treatment. To do this we will ask participants to (1) identify the different health systems they access and (2) explore how participants engage with these health systems for varying health conditions.

Visit 4: Week 24 (Household visit)

Illness narrative follow-up questions (Repeat activity – as per visit 2 and 3)

Household income/expenses activity

Aim: Exploring how income is generated and to explore the effects of TB on household socio-economic status. People living in under-resourced communities experience a greater burden of disease. Through this activity, we explore and illustrate the linkages between socio-economic circumstances and how households manage paediatric TB. This activity should be conducted with the caregiver of the child patient.

1) "*We are going to do a household income/expenses activity. A household income activity includes describing and showing us how money or other resources are brought into and spent from your household. The reason why we want to know this is to help us understand the ways in which RR-TB affects the finances of a household. Your story will help to develop strategies and programmes to better help persons with RR-TB. One way of showing how money or resources is coming into your household is by pretending that all the incomes/expenses are part of a pie/cake. We want to understand what the different 'slices' (incomes) are that form part of the pie/cake.*"
    a. List of all the household income sources and expenses
    b. Effect of the child's TB diagnosis.
    c. How/if their household has been affected by the COVID-19 pandemic/ lockdown measures.

Parameters of ambitions activity

AIM: To understand the parameters of ambition of participants which take the form of hopes, dreams, ambitions, and fears. By discussing these parameters, we may illicit the motivations for general health seeking behaviour such as pursuing/not pursuing health care, adherence, and treatment acceptability.

1) "*We have done several timeline activities about things that have happened in the past. Now we would like to do a timeline about the future*". Ask the participant to draw a timeline (using the standard timeline template) to indicate their plans and dreams for the future of their child (if caregiver) and child (if old enough to participate).

Thank you so much for talking to us. Is there anything else we need to know?

Supplementary File 3: COREQ (COnsolidated criteria for REporting Qualitative research) Checklist

Topic	Item No.	Guide Questions/Description	Reported on Page No.
**Domain 1: Research team and reflexivity**			
Personal characteristics			
Interviewer/facilitator	1	Which author/s conducted the interview or focus group?	Line 172
Credentials	2	What were the researcher's credentials? E.g. PhD, MD	Line 172
Occupation	3	What was their occupation at the time of the study?	Line 172
Gender	4	Was the researcher male or female?	Line 171
Experience and training	5	What experience or training did the researcher have?	Line 172
Relationship with participants			
Relationship established	6	Was a relationship established prior to study commencement?	Line 161
Participant knowledge of the interviewer	7	What did the participants know about the researcher? e.g. personal goals, reasons for doing the research	Line 144
Interviewer characteristics	8	What characteristics were reported about the inter viewer/facilitator? e.g. Bias, assumptions, reasons and interests in the research topic	Line 174
**Domain 2: Study design**			
Theoretical framework			
Methodological orientation and Theory	9	What methodological orientation was stated to underpin the study? e.g. grounded theory, discourse analysis, ethnography, phenomenology, content analysis	Line 108
Participant selection			
Sampling	10	How were participants selected? e.g. purposive, convenience, consecutive, snowball	Line 141
Method of approach	11	How were participants approached? e.g. face-to-face, telephone, mail, email	Line 141
Sample size	12	How many participants were in the study?	Line 207
Non-participation	13	How many people refused to participate or dropped out? Reasons?	n/a
Setting			
Setting of data collection	14	Where was the data collected? e.g. home, clinic, workplace	Line 166
Presence of non-participants	15	Was anyone else present besides the participants and researchers?	Line 165
Description of sample	16	What are the important characteristics of the sample? e.g. demographic data, date	Line 207 - 211
Data collection			
Interview guide	17	Were questions, prompts, guides provided by the authors? Was it pilot tested?	Supp material
Repeat interviews	18	Were repeat inter views carried out? If yes, how many?	Line 167
Audio/visual recording	19	Did the research use audio or visual recording to collect the data?	Line 175
Field notes	20	Were field notes made during and/or after the inter view or focus group?	Line 176
Duration	21	What was the duration of the inter views or focus group?	Line 163
Data saturation	22	Was data saturation discussed?	Line 142
Transcripts returned	23	Were transcripts returned to participants for comment and/or	n/a

Topic	Item No.	Guide Questions/Description	Reported on Page No.
		correction?	
**Domain 3: analysis and findings**			
Data analysis			
Number of data coders	24	How many data coders coded the data?	Line 194
Description of the coding tree	25	Did authors provide a description of the coding tree?	n/a
Derivation of themes	26	Were themes identified in advance or derived from the data?	Line 192
Software	27	What software, if applicable, was used to manage the data?	n/a
Participant checking	28	Did participants provide feedback on the findings?	n/a
Reporting			
Quotations presented	29	Were participant quotations presented to illustrate the themes/findings? Was each quotation identified? e.g. participant number	Lines 207 - 3[illegible]
Data and findings consistent	30	Was there consistency between the data presented and the findings?	Lines 207 - 3[illegible]
Clarity of major themes	31	Were major themes clearly presented in the findings?	Lines 207 - 3[illegible]
Clarity of minor themes	32	Is there a description of diverse cases or discussion of minor themes?	Lines 207 - 3[illegible]

Developed from: Tong A, Sainsbury P, Craig J. Consolidated criteria for reporting qualitative research (COREQ): a 32-item checklist for interviews and focus groups. *International Journal for Quality in Health Care*. 2007. Volume 19, Number 6: pp. 349 – 357
